# Reverse Trigger in Ventilated Non-ARDS Patients: A Phenomenon Can Not Be Ignored!

**DOI:** 10.3389/fphys.2021.670172

**Published:** 2021-07-29

**Authors:** Zhimin Lin, Jing Zhou, Xiaoling Lin, Yingzhi Wang, Haichong Zheng, Weixiang Huang, Xiaoqing Liu, Yimin Li, Nanshan Zhong, Yongbo Huang, Yuanda Xu, Ling Sang

**Affiliations:** ^1^Department of Critical Care Medicine, The First Affiliated Hospital of Guangzhou Medical University, Guangzhou, China; ^2^Guangzhou Institute of Respiratory Health, Guangzhou, China; ^3^State Key Laboratory of Respiratory Disease, Guangzhou, China; ^4^Guangzhou Laboratory, Guangzhou, China

**Keywords:** reverse triggering, mechanical ventilation, sedation, acute respiratory distress syndrome, respiratory mechanics

## Abstract

**Introduction:**

The role of reverse trigger (RT) was unknown in ventilated non-acute respiratory distress syndrome (ARDS) patients. So we conducted a retrospective study to evaluate the incidence, characteristics and physiologic consequence of RT in such population.

**Method:**

Six ventilated non-ARDS patients were included, the esophageal balloon catheter were placed for measurements of respiratory mechanics in all patients. And the data were analyzed to identified the occurrence of RT, duration of the entrainment, the entrainment pattern or ratio, the phase difference (dP) and the phase angle (θ), phenotypes, Effects and clinical correlations of RT.

**Result:**

RT was detected in four patients of our series (66.7%), and the occurrence of RT varying from 19 to 88.6% of their recording time in these 4 patients. One patient (No.2) showed a stable 1:1 ratio and Mid-cycle RT was the most common phenotype. However, the remained patients showed a mixed ratios, and Late RT was the most common phenotype, followed by RT with breath stacking. The average values of mean phase delay and phase angles were 0.39s (0.32, 0.98) and 60.52° (49.66, 102.24). Mean phase delay and phase angles were shorter in early reverse triggering with early and delayed relaxation, and longer in mid, late RT and RT with breath stacking. Pmus was variable between patients and phenotypes, and larger Pmus was generated in Early RT, Delayed Relaxation and mid cycle RT. When the RT occurred, the Peso increased 17.27 (4.91, 19.71) cmH_2_O compared to the controlled breathing, and the average value of incremental ΔPeso varied widely inter and intra patients (Table 3B and Figure 1). Larger ΔPeso was always generated in Early RT, Delayed Relaxation and mid cycle RT, accompanied by an significant increase of PL with 19.12 (0.75) cmH_2_O and 16.10 (6.23) cmH_2_O.

**Conclusion:**

RT could also be observed in ventilated non-ARDS patients. The characteristics of pattern and phenotype was similar to RT in ARDS patients to a large extent. And RT appeared to alter lung stress and delivered volumes.

## Background

Since the research published by *Akoumianaki* (Akoumianaki et al., 2013), the term “reverse trigger (RT)” has attracted the attention of the intensivist in the worldwide (Yonis et al., 2015; Taiga et al., 2018; Takeshi et al., 2018; Bourenne et al., 2019). Reverse trigger is a type of patient-ventilator dyssynchrony whereby a diaphragm muscle contraction occurs after a mandatory breath initiated by the ventilator (Akoumianaki et al., 2013). In Brief, RT indicates an activity of the diaphragm triggered by a ventilator mediated insufflation of the thorax. In a very recent retrospective study (Kassis et al., 2020), Kassis et al. have further described the phenotypes of this phenomenon in details. However, almost all the articles related to RT in critically ill focused on the ventilated acute respiratory distress syndrome (ARDS) patients, and the role of this term in ventilated non-ARDS patients remains unknown. in respiratory physiology, the concept of RT is based on the “respiratory entrainment,” which refers to the establishment of a fixed repetitive temporal relationship between the neural and mechanical respiratory cycles (Simon et al., 1999). Several studies have revealed that the respiratory rhythm can be entrained or phase locked to extrinsic periodic mechanical inflations imposed during controlled mechanical ventilation (Graves et al., 1986; Muzzin et al., 1992; Baconnier et al., 1993). Therefore we purposed that this phenomenon is also available in other ventilated non-ARDS patients, as these patients also interact with the energy system of the ventilator.

We observed the phenomenon of RT in a sedated ventilated lung transplant patient with a continuous esophageal pressure (Pes) recording occasionally. After that, we retrospectively collected and analyzed available data of other ventilated non-ARDS patients. This case series may represent the first description of RT in such population, and this study aims to evaluate the incidence, pattern and phenotypes of RT and explore changes in esophageal pressure (P_*eso*_) and transpulmonary pressure (P_L_) with these efforts in such population.

## Methods

### Study Population

A retrospective study was conducted in a 37 beds general ICU in the 1st affiliated hospital of Guangzhou Medical University. And the analysis was performed on 6 patients from another ongoing trail with acute respiratory failure, which was approved by the Ethics Committee of the First Affiliated Hospital of Guangzhou Medical University (Approval No.2020-065) and the informed consent were obtained from all the patient’s families.

### Data Acquisition

Six patients were ventilated and all of them had an esophageal balloon catheter placed for measurements of respiratory mechanics. DP15 pressure transducers (Validyne Engineering Corp., United States) were respectively connected to the esophageal balloon and the respiratory circuit and were used to record pressures traces (Paw, Peso). The pressure transducers were connected to a CD280 pressure amplifier (Validyne Engineering Corp., United States). The pressure signals ultimately were recorded by PowerLab Data Recording and Analysis system (Powerlab 16/30 SP, ADInstruments Pty Ltd, Australia). The sampling rate was 200 Hz. Transpulmonary pressure (PL) equal to Paw minus Peso. The Paw, Peso, PL were displayed simultaneously. The data were stored in a notebook and were analyzed using commercially available software (labchart7.0, ADinstrucments Pty Ltd, Australia).

### The Definition of Terms

Patient inspiratory effort was detected through the Peso negative fluctuation from baseline. The definition of RT and the following descriptive parameters such as the duration of the entrainment, the entrainment pattern or ratio, the phase difference (dP) and the phase angle (θ) were according to the previous research (Akoumianaki et al., 2013). RT was defined by a sudden negative fluctuation of Peso occurring after the onset of a mandatory breath. RT were divided into 5 phenotypes:(1) Early RT, Early Relaxation; (2) Early RT, Delayed Relaxation; (3) Mid Cycle RT; (4) Late RT and (5) RT with Breath stacking, the definition were according to another previous research (Kassis et al., 2020), and modified through peer-review to clarify definitions.

### Statistical Methods

Data were analyzed by descriptive statistical methods and are expressed as mean ± SD, medians, and the IQR. Statistical analysis was carried out by SPSS 17.0 software.

## Results

### Patient’s Characteristics

Recordings of Pes were available in six patients, who were deeply sedated due to clinical condition. And all of them were ventilated by Evita XL (Dräger), the ventilator mode was either volume assist-control (VAC) or pressure assist-control. More details of patient’s demography, diagnosis, ventilator and respiratory mechanisms were shown in Table 1.

**TABLE 1:** Patient’s demography, diagnosis, ventilator and respiratory mechanics.

Patient No.	Age, years	Gender	Diagnosis	Mode	RR	V_T,_mL/kg IBW	Peak, cm H_2_O	PEEP, cm H_2_O	Timech, s	Crs, mL/cm H_2_0	Rrs, cm H_2_0/L/s,
1	67	F	Interstitial Pneumonia	VAC	16	2.85	20	5	0.95	12.2	14.5
2	30	M	Obliterans Bronchitis	VAC	26	5.6	40	2	0.8	30	35
3	59	F	Bilateral lung transplantation	VAC	16	5.2	18	2	0.9	20.6	13.6
4	75	F	Sepsis	VAC	19	8.4	22	6	0.95	54	14.4
5	58	F	Bilateral lung transplantation	VAC	17	4.1	23	8	0.95	14.8	19.7
6	65	M	Bronchiectasis	VAC	17	6	21	5	1.0	45.9	13.7

*F, female; M, male; PAC, pressure assist-control; VAC, volume assist-control; RR, respiratory rate; V_*T*_, tidal volume; IBW, ideal body weight; PEEP, positive end-expiratory pressure; Timech, mechanical inspiratory time; Crs, respiratory system compliance; Rrs, respiratory system resistance.*

### Reverse Trigger Characteristics

All patients were deeply sedated indicated as RASS score −5. We analyzed waveform data consisted of an average of 7.3 ± 1.14 min per patient for a total of 52.1 min and 971 breaths. Reverse trigger events were detected in 448 total breaths (46.1%) in 4 patients (66.7%) (No. 2, 3, 4, 6), and the occurrence of reverse trigger varying from 19 to 88.6% of their recording time in these 4 patients. Blinded review of phenotypes resulted in 100% agreement, More details were shown in Table 2.

**TABLE 2:** Patient’s sedation level, total recording respirations, reverse trigger duration and ratio, and arterial blood gases results.

Patient No.	RASS	RR_record_	RR_RT_	RR_RT_/RR_record_%	P/F, mm Hg	FiO_2_, %	PH	PaCO_2_	HCO_3_-	BE
1	−5	154	0	0	ECMO support	40	7.449	46.7	32.5	7.6
2	−5	290	257	88.6	204	50	7.379	72.5	41.8	15
3	−5	150	29	19	ECMO support	50	7.391	38.7	27.4	−1.7
4	−5	140	72	51.4	220	55	7.419	44.5	27.2	2.8
5	−5	146	0	0	ECMO support	80	7.416	44.3	27.9	3.6
6	−5	124	82	66.1	298	50	7.473	43	30.9	7.1

*RASS, Richmond Agitation-Sedation Scale; RR_*record*_, Recorded respirations; RR_*RT*_, respirations with reverse trigger; ECMO, extracorporeal membrane oxygenation; P/F, PaO_2_ to FiO_2_ ratio.*

One patient (No.2) showed a stable 1:1 ratio and Mid-cycle RT was the most common phenotype. However, the remained patients showed a mixed ratios, and Late RT was the most common phenotype, followed by RT with Breath stacking, more details were shown in Table 3A and Figures 2, 3.

**FIGURE 1:**
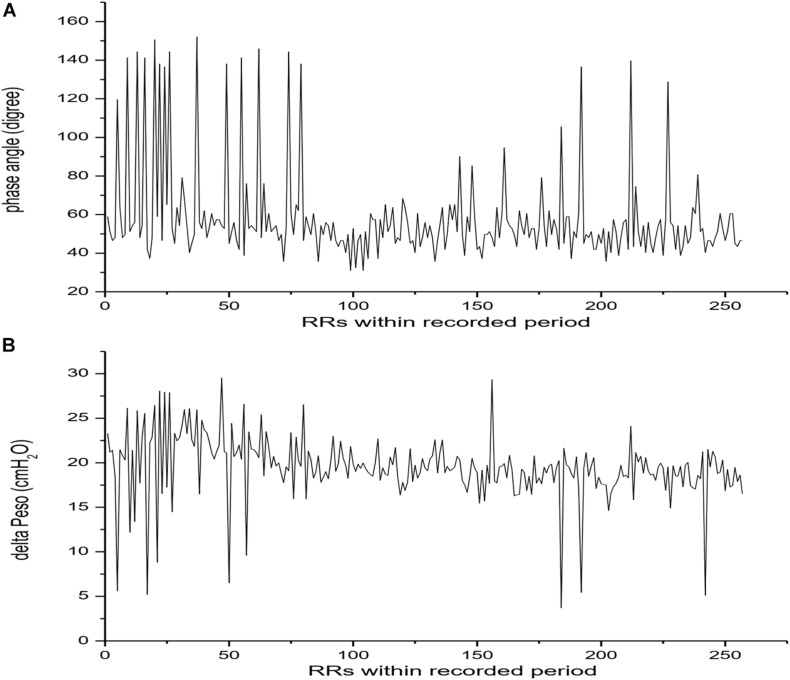
Phase angle and esophageal pressure fluctuation breath by breath in stable reverse trigger epochs acquired in patient 2. Panel **(A)** showed Phase angle fluctuation breath by breath. Panel **(B)** showed the maximal inspiratory muscle pressure fluctuation during reverse trigger breath by breath.

**TABLE 3A:** Phenotypes of reverse trigger.

Patient No.	RTtype	RTtot	Early RT, early relaxation	Early RT, delayed relaxation	Mid Cycle RT	Late RT	Breath stacking
2	Stable(1:1)	257 (100%)	0	19 (7%)	220 (86%)	18 (7%)	0
3	Unstable	29 (100%)	0	0	2 (7%)	19 (65.5%)	8 (27.5%)
4	Unstable	72 (100%)	3 (4.2%)	0	23 (31.9%)	30 (41.7%)	16 (22.2%)
6	Unstable	82 (100%)	0	0	0	57 (69.5%)	25 (30.5%)

*RTtype, type of reverse trigger; RTtot, total numbers of reverse trigger.*

**TABLE 3B:** Phase delay, Phase angle and the ΔPeso during reverse trigger.

Patient No.	Phase delay, dP, s		Phase angle, θ, degree		ΔPeso, cmH_2_O	
	Median	IQR	Median	IQR	Median	IQR
2	0.33	(0.3, 0.38)	51.21	(46.55, 58.97)	19.5	(18.21, 21.12)
3	0.91	(0.89, 0.94)	98.08	(95.93, 101.32)	5.9	(4.22, 7.01)
4	0.96	(0.6, 1.09)	103.87	(65.26, 118.55)	5.24	(4.04, 7.76)
6	1.10	(1.00, 1.13)	104.76	(95.23, 107.62	2.36	(1.30, 4.74)
Average	0.39	(0.32, 0.98)	60.52	(49.66, 102.24)	17.27	(4.91, 19.71)

*ΔPeso, the maximal inspiratory muscle pressure during reverse trigger.*

**FIGURE 2:**
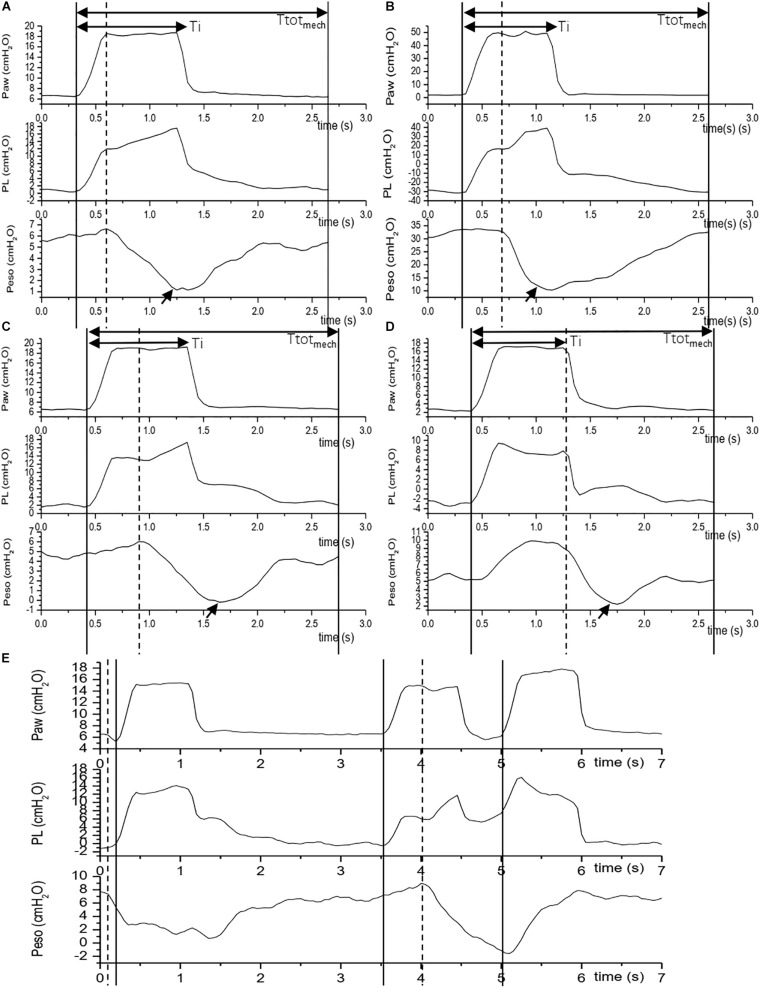
Reverse trigger phenotypes. The figure shows 5 Reverse trigger phenotypes during assist/control ventilation. The vertical solid line indicates an initiation of a mandatory breath, the vertical dashed line indicates an initiation of a patient effort which is express as the negative fluctuation of esophageal pressure, the arrow indicates maximal Inspiratory effort or maximal delta esophageal pressure (Peso). If an initiation of a patient effort precede a mandatory breath, we define it is an assist breath [e.g., the first breath in **(E)**]. If a patient effort emerge after an initiation of a mandatory breath, we define it is a breath with Reverse trigger [e.g., **(A–D)**]. Depending on the time phase of initiation of a patient effort and maximal delta Peso, a Reverse trigger was defined as different phenotypes according to Kassis E B’s classification (Kassis et al., 2020). **(A)** Early RT with Early Relaxation (No.4 patient): patient effort initiation and the max Peso are among inspiratory phase, and termination of patient effort are among early period of expiratory phase; **(B)** Early RT with Delayed Relaxation (No.2 patient): patient effort initiation and the max Peso are among inspiratory phase, and termination of patient effort are among late period of expiratory phase; **(C)** Mid Cycle RT (No.4 patient): patient effort initiate in inspiratory phase and the max Peso emerge in expiratory phase; **(D)** Late RT (No.3 patient): patient effort emerge in expiratory phase; **(E)** RT with breath stacking (No.3 patient): during any phenotype of above reverse trigger, patient effort is sufficient to trigger another assist mechanical breath. Ttot_mech_ is the duration of the mechanical cycle. Ti is the inspiratory phase of the mechanical cycle.

**FIGURE 3:**
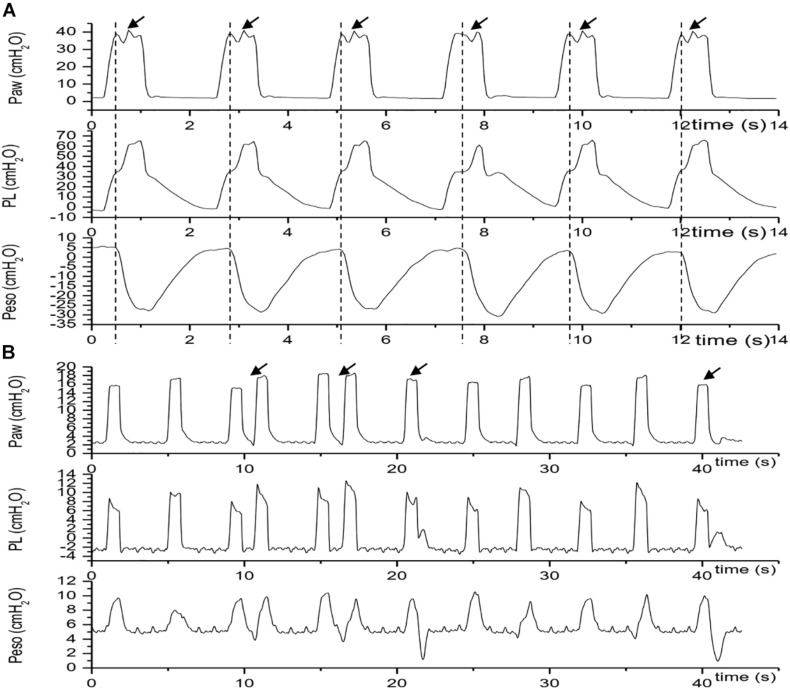
Traces of Paw, PL and Pes during reverse trigger episodes. The vertical dashed line indicates an initiation of a patient effort which is express as the negative fluctuation of esophageal pressure, the arrow indicates a breath with reverse trigger. **(A)** Stable reverse trigger (1:1 entrainment) traces acquired in patient No.2; **(B)** unstable reverse trigger traces acquired in patient No.4. Paw, airway pressure; PL, transpulmonary pressure; Peso, esophageal pressure.

The average values of mean phase delay and phase angles were 0.39s (0.32, 0.98) and 60.52° (49.66, 102.24). In patient No.2, the phase angles varied widely breath by breath (Table 3B and Figure 1). Mean phase delay and phase angles were shorter in early reverse triggering with early and delayed relaxation, and longer in mid, late RT and RT with breath stacking. More details were shown in Table 3C.

**TABLE 3C:** Phase delay, Phase angle and the ΔPeso, PL in each phenotypes of reverse trigger.

	Early RT, early relaxation	Early RT, delayed relaxation	Mid cycle RT	Late RT	Breath stacking
Phase delay, Mean (SD), s	0.38 (0.16)	0.32 (0.04)	0.36 (0.12)	1.04 (0.13)	1 (0.17)
Phase angle, mean (SD), degree	41.33 (16.88)	53 (9.79)	54.51 (17.71)	110.65 (27.02)	101.7 (19.76)
ΔPeso, mean (SD), cmH_2_O	5.44 (0.50)	18.32 (3.36)	18.22 (4.52)	5.66 (7.43)	6.2 (2.29)
ΔPL, mean (SD), cmH_2_O	1.92 (0.64)	19.12 (0.75)	16.10 (6.23)	−0.64 (1.26)	−0.17 (1.90)
PL_*basline*_, mean (SD), cmH_2_O	14.34 (5.89)				

*ΔPeso, the maximal inspiratory muscle pressure during reverse trigger; ΔPL, the maximal inspiratory transpulmonary pressure during the breath with RT minus the maximal transpiratory transpulmonary pressure during the control breath without RT. PL_baseline_, the average value of maximal inspiratory transpulmonary pressure of all analyzed control breath.*

### Reverse Trigger Effects and Clinical Correlations

**FIGURE 4:**
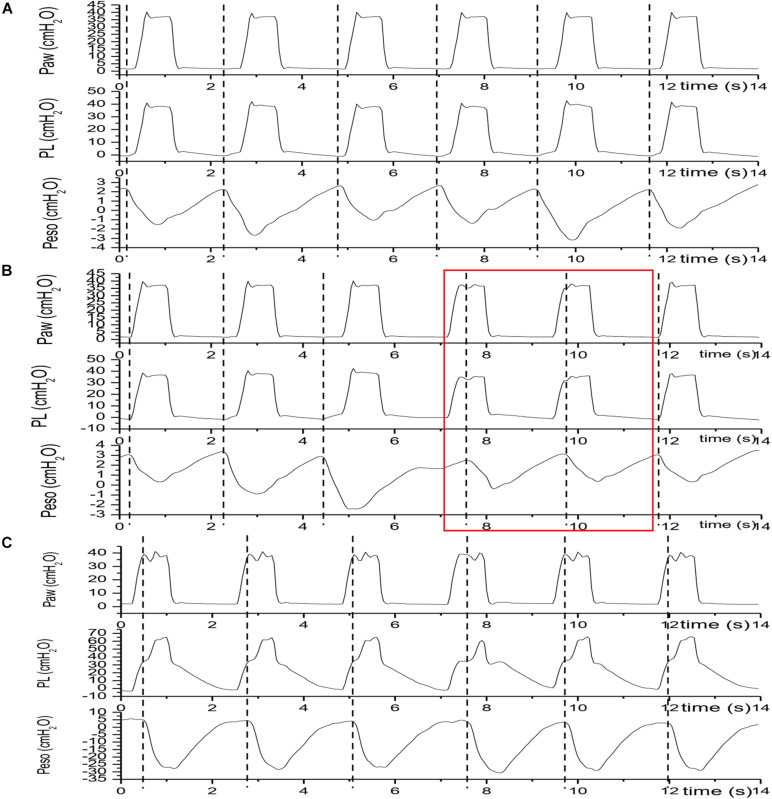
Transition from assist mechanical breath to intermittent (unstable) reverse trigger and stable reverse trigger. Traces were acquired in patient No.2. Dotted line denoted the initiation of each neural breath. Panel **(A)** showed that each neural breath arose before a mechanical breach initiation or the phase delay > 0 (Assist breath); Panel **(B)** showed the coexistence of Assist breath and Control breath with stable reverse trigger. The red solid line box included two Control breath with stable reverse trigger (the 4th and 5th breath). Panel **(C)** showed that each neural breath arose behind a mechanical breach initiation or the phase delay < 0 (Control breath with stable reverse trigger). Paw, airway pressure; PL, transpulmonary pressure; Peso, esophageal pressure.

**FIGURE 5:**
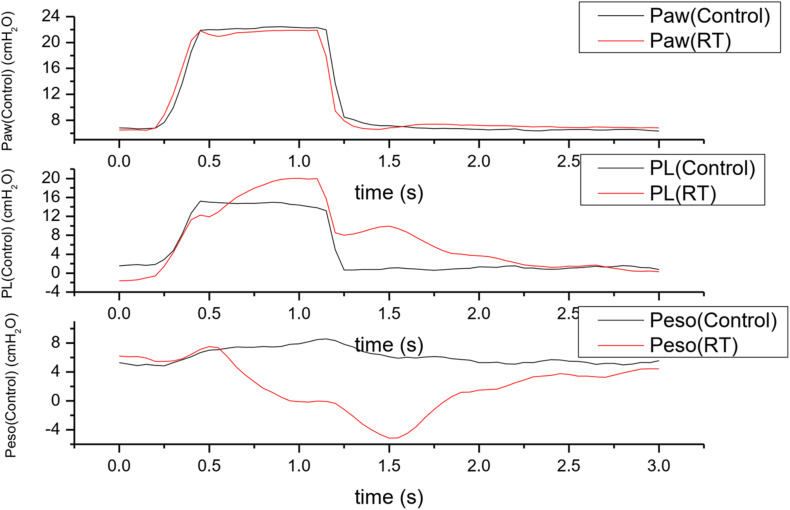
An overlaping display of pressure waveforms of a control breath with reverse trigger and a control breath. Traces were acquired in patient No.4; Paw, airway pressure; PL, transpulmonary pressure; Peso, esophageal pressure. The black solid lines indicates the Paw, PL and Peso fluctuations during a mandatory or control breath. The red solid lines indicates the Paw, PL and Peso fluctuations during a breath with reverse trigger in the same patient. Comparing to the control breath, arising of patient effort or delta Peso may elevates PL during breath with reverse trigger.

The ventilator parameters were not adjusted in entire analysis periods in all patients. in patient No.2, the dosage of sedative drug was decreased in the observational period, even though the RASS score did not change, the patient gradually appeared a transition from assist mechanical breath to intermittent (unstable) RT and stable RT (Figure 4). When the RT occurred, the Peso increased 17.27 (4.91, 19.71) cmH_2_O compared to the controlled breathing, and the average value of incremental ΔPeso varied widely inter and intra patients (Table 3B and Figure 1). Larger ΔPeso was always generated in Early RT, Delayed Relaxation and mid cycle RT, accompanied by an significant increase of PL with 19.12 (0.75) cmH_2_O and 16.10 (6.23) cmH_2_O. More details were shown in Table 3C and Figure 5.

## Discussion

To our knowledge, it is the first study to describe the phenomenon of RT in ventilated non-ARDS patients in ICU. Our study revealed that reverse trigger occurred in 4/6 of our cases, and the phenotypes of RT varied widely, followed by an increment of ΔPeso, especially in Mid cycle RT.

In our study, 2 patients (No.3 and 5) underwent bilateral lung transplantation. Primary graft dysfunction, which was a very common postoperative complication, was similar to ARDS (Talaie et al., 2021). However, these two patients were all on the fifth day after the operation when the esophageal balloon catheter were placed, and the chest X-ray results also showed that the infiltration in both lungs had been obviously absorbed. According to the present knowledge (Talaie et al., 2021) and our clinical practice, we believed that these patients had passed the phrase of PGD, and were readiness for sedation withdrawn and weaning the ECMO. Therefore we still classified them as non-ARDS patients.

Pathophysiologic mechanisms of RT remained controversial. At present the mainstream view of it was in related to the respiratory entrainment (Akoumianaki et al., 2013), which had been reported in animals and healthy subjects (Petrillo et al., 1983; Graves et al., 1986; Muzzin et al., 1992; Simon et al., 1999, 2000). Hering-Breuer reflexes and vagal feedback had been considered essential for entrainment because entrainment was disappeared in anesthetized animals with bilateral vagotomy (Vibert et al., 1981; Petrillo et al., 1983). However, Simon found entrainment in bilateral lung transplant patients. The result of Simon’s trial showed that vagal feedback was not absolutely required for entrainment. Other factors including airflow, temperature, pressure, and airway CO_2_, though upper airway afferents, transferring to the brain may facilitate entrainment (Simon et al., 2000). In the current study, RT could be also detected in lung transplant recipient patients, but we found an unstable pattern of RT in those patients instead of regular entrainment. We speculate that other mechanisms may facilitate RT in addition to entrainment. Kassis et al. (2020) demonstrated that maybe there were other mechanisms, not simply the entrainment. On the other hand, RT was always observed in the deeply sedated ARDS patients who didn’t receive neuromuscular blocking agent in most studies (Akoumianaki et al., 2013; Bourenne et al., 2019; Kassis et al., 2020), which were similar to ours. By contrast, RT occurred in awake ventilated ARDS patients had been reported in two case reports (Yonis et al., 2015; Taiga et al., 2018). In our study we captured a very interesting waveform from No.2 patient, he appeared a transition from assist mechanical breath to intermittent (unstable) RT and stable RT, followed by an increment of ΔPeso and PL, when the sedative drug was reduced, even though the RASS score did not change. We supposed that the proportional relationship between diaphragm contraction force and ventilator energy played the key role in the occurrence of RT, which was in line with the mechanisms of the entrainment. Recently Mellado Artigas et al. (2021) found that the incidence of RT was higher in patients who had more patient-triggered breaths or were more likely to be extubated. Their retrospective results also support our assumption. Interesting we found that three patients (No.1, 3, 5) in our series were with ECMO support, RT was observed in only one patient (No.3), and the occurrence of RT was only 19% in this patient. On the other hand, RT occurred in the rest three patients without ECMO support (No.2, 4, 6), and the occurrence of RT was both higher than patient No.3. As ECMO could control the respiratory drive and effort (Mauri et al., 2016), which meant that it could reduce the strength of diaphragm contraction, the result also supported our previous suppose. Of course our result was not enough to fully answer this question. Actually, it should be resolved by further research in the future.

In the healthy subjects, several studies had demonstrated that stable entrainment ratio such as 1:1 was the most common (Petrillo et al., 1983; Benchetrit et al., 1986; Graves et al., 1986; Rodenstein et al., 1989; Muzzin et al., 1992), which were similar to the results of ventilated ARDS patients (Akoumianaki et al., 2013; Kassis et al., 2020). However, in our study only one patient (No.2) showed stable 1:1 entrainment ratio, and the rest 3 patients showed a state of instability. Obviously it did not prove to be due to the difference between ARDS and non-ARDS patients as the sample size was small. Kassis et al. (2020) demonstrated that RT might also result from an uncoupled underlying respiratory oscillator with efforts independent from the ventilator cycle, which potentially represented a unique etiology rather than entrainment and affect the pattern of RT. However, the potential underlying mechanism of these uncoupled breaths was complex and unclear, future study would be needed to illustrate this problem.

We observed the breathing stacking and elevated PL in the patients when RT occurred. It was in line with the finding of *Kassis’s* research (Kassis et al., 2020). Even though this type of patient–ventilator asynchrony may be harmful and contribute to ventilator induced lung injury (VILI) in ARDS patients (De Haro et al., 2018), the potential role of this phenomenon in patients without ARDS remained unclear. Breath stacking, which would induce a large tidal volume, would cause volutrauma in ARDS patients. What’s more, two system reviews demonstrated that ventilation with lower tidal volumes had a strong potential to prevent development of pulmonary complications and maybe even to improve survival (Fuller et al., 2013; Neto et al., 2014). However, in ventilated patients without ARDS, a low tidal volume strategy did not result in a greater number of ventilator-free days than an intermediate tidal volume strategy (Writing Group for the PReVENT Investigators, Simonis et al., 2018). PL, which represent alveolar distending pressure, was considered to be useful to guide the PEEP titration to keep to lung open in EPVent study (Talmor et al., 2008). In addition, limiting end-inspiratory PL to 20–25 cm H_2_O appears to minimize alveolar overdistention (Grieco et al., 2017). It was obvious that there was a “safe range of PL in ARDS patients. However, in non-ARDS patients, whether this safety range still exist remained unclear. In our study, the average value of maximal PL were 14.34 (5.89) cmH_2_O. The incremental PL could be as high as 19.12 (0.75) cmH_2_O during Early RT with Delayed Relaxation and 16.10 (6.23) cmH_2_O during Mid Cycle RT. Finally due to limited sample size and significant heterogeneity in our data, no definitive recommendations can be made.

Our study had several limitations: (1) the sample size is relatively small, because it is difficult to obtain the complete data in a retrospective study. Besides, Patients included in the current study present with specific traits (three patients supported by ECMO and two lung transplant recipients). Since we observed a high incidence of RT in such population, A prospective study with a larger sample size is necessary to determine the prevalence of RT in Non-ARDS patients. (2) The data was not initiated for this investigation, so we could not clearly illustrate the mechanism, response to intervention and impact on clinical outcome of RT. It was due to nature of the retrospective study and a well-designed prospective study was needed. (3) We did not use Campbell diagram to differentiate the phenotypes of RT, which was used in *Kassis’s* research (Kassis et al., 2020), however, the phenotypes in that study were developed via *post hoc* analysis of existing waveforms, and blinded review of phenotypes via analysis of waveforms resulted in 100% agreement in our study, it increased the credibility of our results.

In conclusion, RT could also be observed in ventilated non-ARDS patients in ICU. The characteristics of pattern and phenotype was similar to RT in ARDS patients to a large extent. And RT appeared to alter lung stress and delivered volumes.

## Data Availability Statement

The original contributions presented in the study are included in the article/supplementary material, further inquiries can be directed to the corresponding author/s.

## Ethics Statement

The studies involving human participants were reviewed and approved by the Ethics Committee of the First Affiliated Hospital of Guangzhou Medical University. Written informed consent for participation was not required for this study in accordance with the national legislation and the institutional requirements.

## Author Contributions

LS and YX had the idea for and designed the study, they had full access to all data in the study and took responsibility for the integrity of the data analysis. LS and JZ wrote the first full draft of the report. All authors contributed to data acquisition, data analysis, or data interpretation, and they all reviewed and approved the final version.

## Conflict of Interest

The authors declare that the research was conducted in the absence of any commercial or financial relationships that could be construed as a potential conflict of interest.

## Publisher’s Note

All claims expressed in this article are solely those of the authors and do not necessarily represent those of their affiliated organizations, or those of the publisher, the editors and the reviewers. Any product that may be evaluated in this article, or claim that may be made by its manufacturer, is not guaranteed or endorsed by the publisher.
